# Serum Ferritin Is Inversely Correlated with Testosterone in Boys and Young Male Adolescents: A Cross-Sectional Study in Taiwan

**DOI:** 10.1371/journal.pone.0144238

**Published:** 2015-12-08

**Authors:** Kuo-Ching Chao, Chun-Chao Chang, Hung-Yi Chiou, Jung-Su Chang

**Affiliations:** 1 Division of Gastroenterology and Hepatology, Department of Internal Medicine, Taipei Medical University Hospital, Taipei, Taiwan; 2 Division of Gastroenterology and Hepatology, Department of Internal Medicine, School of Medicine, College of Medicine, Taipei Medical University, Taipei, Taiwan; 3 School of Public Health, College of Public Health and Nutrition, Taipei Medical University, Taipei, Taiwan; 4 School of Nutrition and Health Sciences, College of Public Health and Nutrition, Taipei Medical University, Taipei, Taiwan; Gentofte University Hospital, DENMARK

## Abstract

**Objective:**

The transition from childhood to teenaged years is associated with increased testosterone and a decreased iron status. It is not clear whether higher testosterone levels cause the decreased iron status, and to what extent, obesity-related inflammation influences the iron-testosterone relationship. The aim of the present study was to examine relationships of testosterone, iron status, and anti-/proinflammatory cytokines in relation to nutritional status in boys and young adolescent Taiwanese males.

**Methods:**

In total, 137 boys aged 7~13 yr were included. Parameters for obesity, the iron status, testosterone, and inflammatory markers were evaluated.

**Results:**

Overweight and obese (ow/obese) boys had higher mean serum testosterone, interleukin (IL)-1β, and nitric oxide (NO) levels compared to their normal-weight counterparts (all *p*<0.05). Mean serum ferritin was slightly higher in ow/obese boys compared to normal-weight boys, but this did not reach statistical significance. A multiple linear regression showed that serum ferritin (β = -0.7470, *p* = 0.003) was inversely correlated with testosterone, while serum IL-10 (β = 0.3475, *p* = 0.009) was positively associated with testosterone after adjusting for covariates. When normal-weight boys were separately assessed from ow/obesity boys, the association between testosterone and serum ferritin became stronger (β = -0.9628, *p*<0.0001), but the association between testosterone and IL-10 became non-significant (β = 0.1140, *p* = 0.4065) after adjusting for covariates. In ow/obese boys, only IL-10 was weakly associated with serum testosterone (β = 0.6444, *p* = 0.051) after adjusting for age.

**Conclusions:**

Testosterone and serum ferritin are intrinsically interrelated but this relationship is weaker in ow/obese boys after adjusting for age.

## Introduction

Associations between androgens and erythropoiesis have been known for more than half a century [1]. Low testosterone levels are a potential risk factor for anemia in older men and women [2]. In particular, hypogonadal men have a 5-fold (1.41~21.8) higher risk of anemia compared to eugonadal men [2]. Testosterone administration to hypogonadal men induces erythropoiesis via increased erythropoietin (EPO) and inhibited hepcidin levels [3,4]. Low hepcidin, a key regulator of iron metabolism, leads to a higher iron absorption rate in the small intestine. EPO can increase iron incorporation into red blood cells (RBCs) in the bone marrow [5]. It is also recognized that iron may exert specific effects on androgen. For example, a pituitary iron overload predicts hypogonadism in thalassemia patients with transfusional iron overload [6]. Liver iron overload is associated with increased sex hormone-binding globulin (SHBG) and moderate hypogonadotropic hypogonadism in men with non-genetically dysmetabolic iron overload syndrome (DIOS) [7]. Eugonadal men with iron-deficiency anemia (IDA) who received intravenous iron therapy (800~1200 mg elemental iron) for 12 weeks exhibited increased levels of testosterone, luteinizing hormone (LH), follicle-stimulating hormone (FSH), and sperm parameters [8].

Obesity is frequently associated with low testosterone [9] and high serum ferritin levels [10]. Both testosterone and iron may interact with inflammatory responses. Testosterone can suppress proinflammatory responses but upregulates immunomodulatory cytokines such as interleukin (IL)-10 [11,12]. Proinflammatory cytokines are potent regulators of serum ferritin and hepcidin. Hepcidin plays a key role in the innate and adaptive immunities [13]. Elevated serum ferritin can function as a proinflammatory modulator by upregulating IL-1β, tumor necrosis factor (TNF)-α, and nitric oxide (NO) transcriptional activity [14,15].

The transition from childhood to teenaged years is associated with increased testosterone and a decreased iron status. Currently, it is not clear whether higher testosterone levels cause the decreased iron status, and to what degree obesity-related inflammation influences the iron-testosterone relationship in young boys. The broad aims of this study were: 1) to investigate the relationship between testosterone and the iron status in terms of the nutritional status; and 2) to evaluate the effects of anti-/proinflammatory cytokines on testosterone levels in boys and young adolescent males.

## Materials and Methods

### Study participants

In total, 137 (71 normal-weight and 66 overweight and obese (ow/obese)) boys were included in the analysis: 36 boys were aged 7.43±0.56 yr (20 normal weight and 16 ow/obese), 46 boys were aged 10.68±0.51 yr (27 normal weight and 19 ow/obese), and 56 young adolescents were aged 13.11±1.08 yr (23 normal weight and 33 ow/obese). The study was approved by the Research Ethics Committee of Taipei Medical University (201204011). Informed parental written consent was obtained before enrollment in the study.

### Data collection

Details of data collection were previously described elsewhere [16]. Age- and sex-specific cutoff points for the body-mass index (BMI) were used to define overweight and obesity in boys and adolescent males according to guidelines of the Department of Health, Taiwan (Table 1) [17,18]. The BMI was calculated as the mass (kg)/[height (m)]^2^.

**Table 1:** Age- and gender-specific cutoff points for the body-mass index (BMI) for overweight and obese boys and young adolescents according to guidelines of the Department of Health, Taiwan.

		BMI (kg/m^2^)	
Age (years)	Normal	Overweight	Obese
7	14.7~18.5	≥18.6	≥21.2
8	15.0~19.2	≥19.3	≥22.0
9	15.2~19.6	≥19.7	≥22.5
10	15.4~20.2	≥20.3	≥22.9
11	15.8~20.9	≥21.0	≥23.5
12	16.4~21.4	≥21.5	≥24.2
13	17.0~22.1	≥22.2	≥24.8

### Blood biochemical assessment

Fasting blood samples were collected in vacuum tubes containing EDTA. All blood samples were separated into RBCs and serum, and stored at -80°C until being analyzed. Serum IL-1β, interferon (IFN)-γ, and IL-10 levels were determined by enzyme-linked immunosorbent assay (ELISA) kits (Procarta Cytokine Assay Kit; Affymetrix, Santa Clara, CA, USA) according to the manufacturer’s instructions. As an indicator of NO production, the nitrite concentration in the serum was determined with the Griess reagent (Sigma-Aldrich, St. Louis, MO, USA). Serum hepcidin was assessed by an ELISA (DRG International, Marburg, Germany). Serum ferritin was measured using a commercially available electrochemiluminescence immunoassay and was quantitated with a Roche Modular P800 analyzer (Mannheim, Germany). Serum iron and the total iron-binding capacity (TIBC) were measured by a ferrozine-based colorimetric method. The percent of transferrin saturation (%TS) was calculated by [serum iron/TIBC] x 100%. Serum testosterone was measured by an electrochemiluminescence immunoassay and was quantitated by a Modular analytics cobas E601 analyzer (Roche).

### Statistical analysis

Statistical analyses were performed using the Statistical Analysis Systems software (SAS vers. 9.22; SAS Institute, Cary, NC, USA). Continuous data are presented as the mean±standard deviation (SD) and were assessed by an unpaired Student’s *t*-test. Variables not normally distributed were natural log-transformed to achieve a normal distribution and to allow the use of parametric tests. Associations between the serum testosterone concentration and other laboratory parameters were assessed using Pearson’s rank correlation coefficients. A multivariate linear regression model was used to examine relationships between the dependent variable (serum testosterone) and potential variables including age, BMI, iron parameters, and inflammatory cytokines. *p*<0.05 was considered statistically significant.

## Results

### Baseline characteristics

In total, 137 boys participated in this study. The mean age was 10.48±0.26 yr and the mean BMI was 20.2±4.1 kg/m^2^. The mean serum testosterone was 4.1±5.9 nmol/L, and mean serum ferritin was 151.9±130.3 pmol/L. Ow/obese boys had higher serum testosterone concentrations compared to their normal-weight counterparts (Table 2). The mean serum ferritin was slightly higher in ow/obese boys compared to normal-weight boys, but this did not reach statistical significance (Table 2). There were no significant differences in age, serum iron, TIBC, %TS, hepcidin, IFN-γ, or IL-10 between normal weight and ow/obese boys (Table 2). Compared to their normal-weight counterparts, ow/obese boys had higher levels of IL-1β and NO (both *p*<0.05; Table 2).

**Table 2:** Clinical and biochemical data according to the nutritional status (*N* = 137).

Variable ^a^	Boys (*N* = 137)		
	Normal (*n* = 71)	Ow/obese (*n* = 66)	*p* value ^b^
Age (yr)	10.13 (0.28)	10.82 (0.30)	0.093
Body-mass index (kg/m^2^)	17.20 (2.10)	24.30 (5.60)	<0.0001
Log serum iron (μmol/L)	0.79 (0.01)	0.80 (0.01)	0.694
Log serum TIBC (μmol/L)^c^	1.04 (0.00)	1.04 (0.00)	0.960
Log serum ferritin (pmol/L)	8.89 (0.18)	9.32 (0.13)	0.068
Log transferrin saturation (%)	3.24 (0.05)	3.27 (0.05)	0.701
Log hepcidin (ng/ml)	4.43 (0.08)	4.29 (0.10)	0.291
Log interleukin-1β (pg/ml)	0.11 (0.08)	0.49 (0.46)	0.044
Log interferon-γ (pg/ml)	1.55 (0.09)	1.46 (0.78)	0.554
Log nitric oxide (μM)	1.40 (0.11)	1.97 (0.09)	0.031
Log interleukin-10 (pg/ml)	1.44 (10.24)	1.07 (10.19)	0.401
Log testosterone (nmol/L)	0.10 (0.02)	0.50 (0.08)	0.005

^a^ Mean (standard deviation).

^b^ According to an unpaired Student’s *t*-test.

^c^ TIBC, total iron-binding capacity.

Ow, overweight.

### Distributions of testosterone, iron parameters, and cytokines in relation to age and the nutritional status

**Fig 1:**
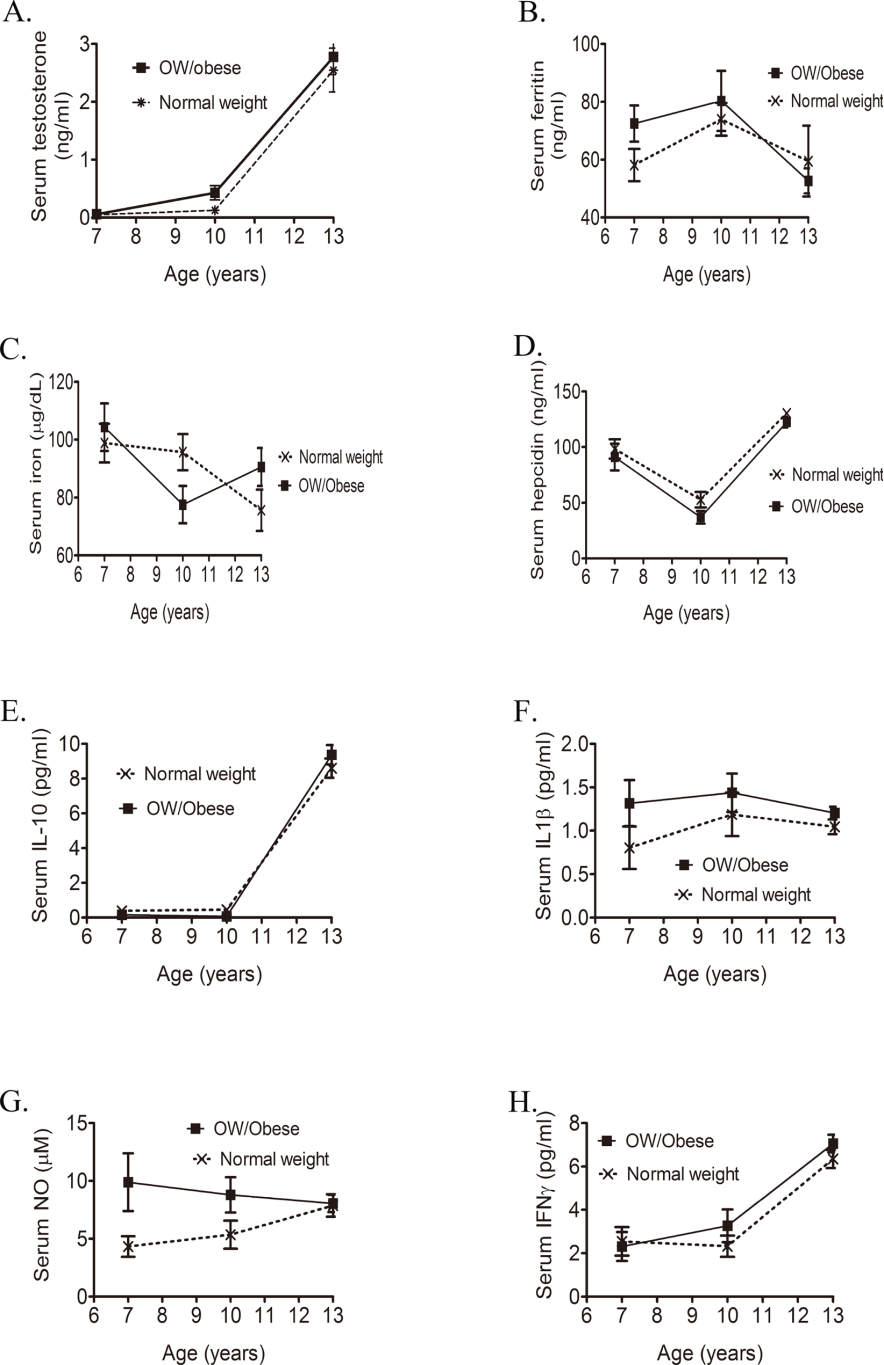
Distributions of serum testosterone (A), ferritin (B), iron (C) hepcidin (D), interleukin (IL)-10 (E), IL-1β (F), nitric oxide (NO) (G), and interferon (IFN)-γ (H) stratified by age and the body-mass index (BMI) (*n* = 137).

We next evaluated distributions of testosterone, iron parameters, and inflammatory cytokines stratified by age and BMI (Table 3). Distributions of serum testosterone (A), IL-10 (E), and IFN-γ (H) were positively associated with age and, to a lesser extent, BMI (Fig 1). In contrast, serum ferritin and serum iron concentrations sharply decreased in those aged 13 yr (Fig 1B and 1C). A V-shaped hepcidin curve was found in both normal-weight and ow/obese boys (Fig 1D). Distributions of serum IL-1β (F) and NO (G) remained stable during the transition from childhood to teenaged years (Fig 1).

**Table 3:** Biochemical characteristics of study participants according to their age and nutritional status.

**Variable** ^**a**^	**Boys**	**Age (years)**		
		7.4 (0.6)	10.7 (0.5)	13.1 (1.1)
BMI (kg/m^2^)	normal	15.5 (1.9)	17.2 (2.2)	18.3 (3.5)
	ow/obese ^b^	20.6 (3.1)***	23.1 (3.8)***	25.0 (4.6)***
Serum iron (μmol/L)	normal	16.3 (1.2)	17.2 (1.2)	13.5(1.3)
	ow/obese	18.7 (1.5)	14.4 (1.1)	16.2 (1.2)
Serum ferritin (pmol/L)	normal	130.8 (12.6)	166.1 (13.3)	133.7 (27.6)
	ow/obese	162.9 (14.2)	186.1 (24.0)	118.4 (9.7)
Transferrin saturation (%)	normal	29.9 (1.8)	31.0 (2.0)	22.1 (2.3)
	ow/obese	33.3 (2.7)	25.6 (2.1)	27.5 (2.1)
Hepcidin (ng/ml)	normal	98.3 (8.6)	56.7 (6.6)	130.5 (4.0)
	ow/obese	91.1 (12.0)	46.0 (6.1)	122.5 (4.8)
Testosterone (nmol/L)	normal	0.2 (0.0)	0.4 (0.1)	8.8 (1.3)
	ow/obese ^b^	0.2 (0.1)	1.4 (0.5)*	9.6 (1.1)
NO (μM)	normal	4.3 (0.9)	5.6 (1.2)	7.9 (1.0)
	ow/obese	9.9 (2.5)	7.8 (1.2)	8.1 (0.7)
IL1β (pg/ml)	normal	0.8 (0.2)	1.1 (0.2)	1.0 (0.1)
	ow/obese	1.3 (0.3)	1.4 (0.2)	1.2 (0.1)
IFNγ (pg/ml)	normal	1.2 (0.4)	3.1 (0.7)	17.9 (1.5)
	ow/obese ^b^	1.1 (0.4)	2.3 (0.6)	25.6 (1.7)**
IL10 (pg/ml)	normal	0.39 (0.12)	0.43 (0.13)	8.6 (0.6)
	ow/obese ^b^	0.23 (0.13)	0.08 (0.05)*	9.4 (0.6)

^a^ Mean (standard deviation).

^b^ Unpaired student’s *t*-test for comparing normal and overweight (ow)/obese boys in the same age group

* *p*<0.05

** *p*<0.01

*** *p*<0.001.

### Serum ferritin is independently associated with testosterone in normal-weight boys

Pearson’s rank correlations analysis showed a strong positive correlation between serum testosterone and IL-10 (*r* = 0.3082), and a significant inverse relationship between serum testosterone and serum ferritin (*r* = -0.2821) after adjusting for age and the BMI (Table 4, adjusted; both *p*<0.01). We next performed a multiple linear regression analysis to predict variants that were independently associated with testosterone concentrations. After adjusting for covariates, serum ferritin (β = -0.7470, *p* = 0.0003) was inversely correlated with testosterone, while serum IL-10 (β = 0.3475, *p* = 0.009) was positively associated with testosterone (Table 5, pooled, multivariant). When normal-weight boys were assessed separately from ow/obese boys, the association between testosterone and serum ferritin (β = -0.9628, *p*<0.0001) became stronger after adjusting for covariates (Table 5, normal weight, multivariant). However, the association between testosterone and IL-10 (β = 0.1140, *p* = 0.4065) became non-significant after adjusting for age and serum ferritin. In ow/obese boys, only IL-10 was weakly associated with serum testosterone (β = 0.6444, *p* = 0.051) after adjusting for age (Table 5, ow/obese).

**Table 4:** Pearson’s rank correlation coefficient and partial *r* of log-transformed serum testosterone with selected iron statuses and inflammatory cytokines in 137 boys.

Variable	Boys (log testosterone)			
	Crude		Adjusted*	
	*r*	*p* value	*r*	*p* value
Age	0.7789	<0.0001	-	-
Log serum iron (μmol/L)	-0.0912	0.289	0.1403	0.103
Log serum TIBC (μmol/L)	0.1981	0.020	0.0749	0.385
Log serum ferritin (pmol/L)	-0.3458	<0.0001	-0.2821	0.001
Log transferrin saturation (%)	0.1054	0.221	0.1054	0.221
Log hepcidin (ng/ml)	0.3399	0.0001	0.1547	0.092
Log interleukin-1β (pg/ml)	-0.1080	0.252	-0.035	0.712
Log interferon-γ (pg/ml)	0.3881	<0.0001	-0.076	0.413
Log nitric oxide (μM)	0.0560	0.533	-0.1074	0.233
Log interleukin-10 (pg/ml)	0.7501	<0.0001	0.3082	0.003

*Adjusted for age and the body-mass index.

TIBC, total iron-binding capacity.

**Table 5:** Multivariate regression coefficients for log-transformed serum testosterone in relation to the nutritional status in 137 boys.

**Pooled**	**Crude**		**Age-adjusted**		**Multivariant** *	
	β	*p* value	β	*p* value	β	*p* value
Log serum iron (μmol/L)	-0.4765	0.289	0.4725	0.103		
Log serum TIBC (μmol/L)	2.7230	0.020	0.6588	0.385		
Log serum ferritin (pmol/L)	-1.0990	<0.0001	-0.5700	0.001	-0.7470	0.0003
Log transferrin saturation	-0.7537	0.071	0.3351	0.221		
Log hepcidin (ng/ml)	0.9321	0.0001	0.2767	0.092		
Log interleukin-1β (pg/ml)	-0.4033	0.252	-0.0863	0.712		
Log interferon-γ (pg/ml)	1.02901	<0.0001	-0.1507	0.712		
Log nitric oxide (μM)	0.1311	0.533	-0.1584	0.233		
Log interleukin-10 (pg/ml)	0.9667	<0.0001	0.4215	0.003	0.3475	0.009
**Normal weight**	**Crude**		**Age-adjusted**		**Multivariant** *	
	β	*p* value	β	*p* value	β	*p* value
Log serum iron (μmol/L)	-0.8392	0.133	0.31849	0.391		
Log serum TIBC (μmol/L)	3.5131	0.029	1.4981	0.151		
Log serum ferritin (pmol/L)	-1.2020	<0.0001	-0.8419	<0.0001	-0.9628	<0.0001
Log transferrin saturation	-1.1557	0.029	0.1259	0.729		
Log hepcidin (ng/ml)	1.3455	0.003	0.5173	0.079		
Log interleukin-1β (pg/ml)	-0.7039	0.131	-0.4281	0.166		
Log interferon-γ (pg/ml)	0.6818	0.058	-0.2466	0.316		
Log nitric oxide (μM)	0.3145	0.268	-0.2466	0.316		
Log interleukin-10 (pg/ml)	0.8792	<0.0001	0.3249	0.042	0.1140	0.4065
**Overweight and obese**	**Crude**		**Age-adjusted**		**Multivariant**	
	β	*p* value	β	*p* value	β	*p* value
Log serum iron (μmol/L)	-0.0432	0.951	0.6006	0.189		
Log serum TIBC (μmol/L)	2.0027	0.220	-0.0153	0.988		
Log serum ferritin (pmol/L)	-1.0795	0.0272	-0.0899	0.790		
Log transferrin saturation	-0.3287	0.601	0.4893	0.236		
Log hepcidin (ng/ml)	0.8427	0.002	0.2598	0.195		
Log interleukin-1β (pg/ml)	-0.1064	0.843	0.2813	0.430		
Log interferon-γ (pg/ml)	1.3770	<0.0001	0.1391	0.623		
Log nitric oxide (μM)	-0.2278	0.470	-0.3408	0.091		
Log interleukin-10 (pg/ml)	1.0900	<0.0001	0.6444	0.051		

^**#**^ Overweight and obese: body-mass index of ≥85th percentile of the age- and Sex-specific value.

* Multivariate model adding age, serum ferritin, and interleukin-10.

## Discussion

Our study indicated that testosterone and serum ferritin are intrinsically interrelated, but this relationship became weaker in ow/obese boys after adjusting for age. It has long been speculated that sex hormones may interact with iron at the systemic level, but the effects of obesity on this relationship are not clear. Obesity is associated with decreased serum testosterone but increased serum ferritin levels [7]. Elevated serum ferritin, an acute-phase reactant, is strongly associated with central obesity and metabolic syndrome [10,19–21]. A recent study involving 1999 healthy Chinese adult men showed that serum ferritin levels were inversely correlated with testosterone, free testosterone, and SHBG levels [22]. Our study in normal-weight boys and adolescent males in Taiwan confirmed this relationship. Other studies showed that serum ferritin levels significantly decreased in elderly obese hypogonadal men who received testosterone therapy [3,4]. These data suggest that testosterone exerts a direct regulatory function on ferritin synthesis, and decreased testosterone may lead to higher serum ferritin levels in obese men. Whether elevated serum ferritin further downregulates testosterone synthesis remains unclear. Overall, our study, together with others, suggests that the testosterone-ferritin axis may play an important role in maintaining physiological androgen function in boys.

Our study is in agreement with results reported in elderly men in whom testosterone and iron levels are closely associated [3]. Aging may affect this relationship, but the mechanisms underlying age-related differences in the erythropoietic response to testosterone are unknown [23,24]. Elderly men experience a decline in testosterone and iron levels and pathophysiological changes that may accompany this decline. The presence of chronic inflammation leads to elevated serum hepcidin levels and anemia of chronic inflammation in the elderly [25]. In addition, aging also affects hemopoietic stem cell production and the endocrine milieu (e.g., EPO secretion) [25]. Coviello and colleagues compared the effects of testosterone therapy on erythropoiesis in young and older men and reported that testosterone-induced increases in the hemoglobin (Hb) and hematocrit levels are more pronounced in older men [23]. However, the greater increase in the Hb level observed in older men during testosterone therapy was not explained by changes in EPO [23]. Interestingly, Bachman *et al*. showed that greater increases in Hb and hematocrit levels in older men during 20 weeks of testosterone therapy were related to greater suppression of serum hepcidin levels in older men than in young men [24]. In our study, the crude analysis of pooled samples showed a significant positive association between testosterone and hepcidin levels, but this relationship became non-significant after adjusting for age. When elementary school boys (aged 7 and 10 yrs) were separately from junior high school (aged 13 yrs), a significant inverse relationship between testosterone and hepcidin was found in elementary school boys (*r* = -0.405; *p* = 0.0027), which remained significant after adjusting for age and BMI (*r* = -0.376; *p* = 0.048) (data not shown). No significant difference was found in junior high school boys (*r* = 0.126; *p* = 0.623). Overall, these data suggest that the relationship between testosterone and hepcidin is age-related, and biological changes that occur during puberty may transiently alter this relationship.

Our study found a positive relationship between IL-10 and testosterone. We hypothesized that the effect of IL-10 on testosterone might not be direct, but rather, indirect via interacting with serum ferritin. The literature suggests that the interaction between serum ferritin and IL-10 is bidirectional. The ferritin H chain was shown to inhibit the immune response of lymphocytes through inducing IL-10 production [26]. However, excess IL-10 may also cause hyperferritinemia. An *in vitro* study showed that recombinant IL-10 treatment directly stimulated ferritin translation in human monocytic cells [27]. A human study reported that IL-10 supplementation is associated with increased risks of hyperferritinemia and anemia in Crohn’s disease patients [27]. On the other hand, sickle cell anemia patients with iron overload, defined by elevated serum ferritin of >2247 pmol/L, had lower serum IL-10 levels compared to non-iron-overloaded patients [28]. Future studies investigating the interactive effects of IL-10 and serum ferritin on testosterone are needed in order to understand how a shift in the anti-/proinflammatory balance contributes to testosterone levels in boys and adult men.

Measuring hepcidin in biological fluids has been difficult [29]. In addition, differences in methodology and the lack of normal reference ranges for serum hepcidin hamper the use of hepcidin as a diagnostic tool and therapeutic target [30]. Mass spectrometry (MS) [31] and immunological-based assays such as ELISA [32] are two of the most-often used methods to analyze serum hepcidin levels. The circulating bioactive form of hepcidin is a small 25-amino-acid (aa) peptide. Being a small peptide, it is difficult to raise antibodies against it. The advantage of MS-based platforms is that they are able to discriminate between the bioactive 25-aa form and other smaller bioinactive isoforms (e.g., 22- and 20-aa peptides) [33]. However, MS-based assays require expensive equipment that is not widely available. According to literature reports [30,34,35], MS- and ELISA-based detecting methods yield similar results in terms of analytical variations and between-sample variations. However, some authors also observed that immunological assays tend to yield higher concentrations of hepcidin than do MS methods. This can be due to either (1) differences in the internal and external standards used by the different methods or (2) the concomitant detection of both the bioactive form of hepcidin-25 and bioinactive isoforms of hepcidin-20 and -22 by the ELISA assay. Our study used a commercially available hepcidin ELISA kit from DRG International, which is based on the principle of competitive binding. Therefore, our assay excluded prohepcidin (the 60-aa premature form of hepcidin), but may also detect isoforms hepcidin-20 and -22 in addition to hepcidin-25. The immunological assay offers a simple, accurate, and reproducible method for detecting serum hepcidin levels. Future studies on large subsets from general populations are recommended in order to establish reliable reference ranges of serum hepcidin concentrations for clinical diagnoses.

Data on obesity and androgen levels in children and adolescent boys are scarce and inconsistent [36]. Hence, causal relationships between obesity and androgen levels remain undefined. Some studies showed that obese boys had lower SHBG and total testosterone compared to normal-weight boys [36,37], but another study revealed elevated testosterone in obese children [9]. In our study, ow/obese boys had higher total testosterone levels than normal-weight boys. Testosterone is an important regulator of the body composition, particularly muscle mass and fat mass [38]. Elderly men with a low to normal gonadal status that received testosterone supplementation for 1 yr showed increased muscle mass and decreased fat mass compared to those who received a placebo [38]. Wabitsch and colleagues first demonstrated that the testosterone level is negatively associated with serum leptin in boys, and the addition of testosterone to human primary adipocytes reduced leptin secretion by up to 62% compared to a control [39]. Later, Soderberg *et al*. further suggested that the negative influence of testosterone on leptin production is lost with increasing adiposity [40]. These data suggest that testosterone is an important regulator of central adiposity, and decreased testosterone may increase adiposity in obese individuals.

There are several limitations to our study which need to be taken into account when interpreting the results. The small sample size and the cross-sectional nature of the study are two limitations. In order to understand the causal relationship between androgen and the iron status, a longitudinal study is needed to determine if changes in serum ferritin concentrations over time predict testosterone levels in boys. A follow-up study will also help clarify the interactive effect of serum ferritin and obesity-related inflammation (e.g., IL-10) on testosterone expression in boys. Our study did not assess the pubertal status and only measured total testosterone due to time and budget constraints. The pubertal status is known to affect testosterone levels and the iron status. Despite the relative small sample size and the lack of information on pubertal development and other sex steroid hormones, we still observed a significant inverse relationship between testosterone and serum ferritin in boys. This suggests there are strong cross-talk signals between sex hormones and ferritin at the systemic level.

## Conclusions

Overall, our study results suggest that serum ferritin independently predicted testosterone levels but this relationship became weaker in ow/obese boys after adjusting for age. Understanding the interactive relationship between serum ferritin and testosterone may help clarify the etiology of obesity-related hypogonadism.

## Abbreviations

BMI: body-mass index
DIOS: dysmetabolic iron overload syndrome
EPO: erythropoietin
FSH: follicle-stimulating hormone
IDA: iron-deficiency anemia
IFN-γ: interferon gamma
IL-1β: interleukin-1β
IL-10: interleukin-10
LH: luteinizing hormone
NO: nitric oxide
ow/obese: overweight and obese
RBCs: red blood cells
SF: serum ferritin
SHBG: sex hormone-binding globulin
TIBC: total iron-binding capacity
TNF-α: tumor necrosis factor-α
%TS: percent transferring saturation
